# Assessment of Microstructural Features of a Silchrome 1 Exhaust Valve of a Harley-Davidson WLA World War II Motorcycle

**DOI:** 10.3390/ma17164156

**Published:** 2024-08-22

**Authors:** Jan Růžička, Ali Alıcıoğlu, Jérémie Bouquerel, Pavel Novák, Jean-Bernard Vogt

**Affiliations:** 1 Department of Metals and Corrosion Engineering, Faculty of Chemical Technology, University of Chemistry and Technology Prague, 166 28 Prague, Czech Republic; paja.novak@vscht.cz; 2 Univ. Lille, CNRS, INRAE, Centrale Lille, UMR 8207—UMET—Unité Matériaux et Transformations, F-59000 Lille, France; jeremie.bouquerel@centralelille.fr; 3 Department of Materials Engineering, TUM School of Engineering and Design, Technical University of Munich, 85748 Munich, Germany; ali.alcoglu@tum.de

**Keywords:** forensic metallurgy, historical metallurgy, silchrome 1, X45CrSi9-3, EN 52 steel, silchrome, poppet valve, heat resistant steel, combustion engine, heat treatment

## Abstract

The paper aims at documenting the material employed in 1942 for the fabrication of an exhaust valve for a Harley-Davidson WLA/WLC motorcycle and assesses the material features with modern steel standard specifications and treatment. Facing properties of the original historical parts of technical heritage objects according to modern standards is a rare discipline, as these objects are nowadays in collections of museums or private collectors and experimental instrumental analyses are strictly forbidden. In this case, a preserved accessible unused surplus replacement kit was studied. The microstructure was assessed by light optical and scanning electron microscopy, electron probe micro-analysis and by heat treatment–hardness correlation. It was found that the valve was made of Silchrome 1 steel in coherence with the X45CrSi9-3 steel modern material standard, but with a slightly higher content of phosphorus and sulfur. Microscopic observations and hardness profile testing suggested a tempered martensitic structure (sorbite) with very fine grains uniformly distributed in the valve and an even heat treatment. Heat treatment–hardness experimentation demonstrated that the original heat treatment cannot be achieved by the modern standard procedure. The tempering temperature was surprisingly deduced to be lower than the recommended one according to the modern standard, which contrasts with the service temperature indicated in the contemporary motorcycle mechanics handbook.

## 1. Introduction

The motorcycle, invented in 1894, has become a very popular means of transport for leisure traveling as well as for daily commuting worldwide. During the last 51 years of motorcycle studies in the Asian region, among other topics, motorcycle engineering and performance as well as advanced technologies have been elaborated upon. However, in the last twenty years, emissions and environmental impacts including urban sustainability have become highly important fields of interest [1]. Therefore, future research is heading toward technology-based driving safety and eco-friendly fuels becoming hot topics [1]. This, of course, supposes an evolution of the materials employed for the different components, including engines. In the European field in the last decade, sealing of the combustion chamber has been discussed intensively in relation to emission limits and Euro standards. Electro mobility and phasing out of combustion engines in general are the hot topic nowadays across a large scope. Besides electric vehicles, a different trend responding to the question of replacing oil-based fuel engines is the use of hydrogen as a fuel in modified internal combustion engines with a conventional layout [2]. Hydrogen combustion engines are no longer sci-fi, as Toyota, Mazda, BMW and Chevrolet vehicles’ power units were made numbering in the dozens before year 2010 [3]. This implies that the hydrogen combustion process will be different from the oil-based fuel process, and suggests taking into consideration all the developments of power units of last 150 years, for which, indeed, valves are compulsory components.

Long-durability and high-reliability valves must be manufactured to avoid valve failures. Cyclic thermal and mechanical loading combined with an aggressive environment are the main sources of their damage. Johnson et al. [4] summarized the cause, effect and avoidance of the valve failure, while significant number of issues can be eliminated by strengthening the basic material [4]. That means changing the material composition, referring to the use of another steel grade or other non-steel material. Acceptable valve quality using non-steel materials is reflected by the high costs of a single product [5]. However, in the mass production of relatively cheap valves, economic aspects including the raw material, the continuous maintenance of tooling and replacement according to shaping process parameters make the “traditional” steels still attractive [6]. Moreover, steels can be easily surface-treated to better resist aggressive environments [7].

Development of martensitic stainless steel after its discovery in 1915 was obviously very attractive for valve fabrication, thanks to its resistance to corrosion, wear, mechanical loading and its possible high-temperature application. In general, martensitic stainless steels typically contain 12–17 wt% Cr, 0–4 wt% Ni and 0.1–1.2 wt% C. For the required properties, such as improved corrosion resistance, machinability, toughness, etc., other alloying elements can be added, including Mo, V, Nb, Al or N. Focusing on the understanding of the microstructure resulting from heat treatment, chemical composition and the forming process allows for very high-performance martensitic steels [8]. The martensitic stainless steel with a commercial denomination “Silchrome 1” is suspected to be the investigated material of this study, as it was employed as Harley-Davidson WLA/WLC World War II motorcycle exhaust valve material. The “W” designation indicates a 45 cubic inch flathead engine, “L” indicates high compression by contemporary standards, “A” indicates army model and “C” indicates Canadian Army model. Silchrome 1 (C 0.40–0.50, Si 2.70–3.30, Mn 0.50–1.50, P max 0.040, S max 0.030, Cr 8.00–10.00, Ni max 0.50 wt%, [9]) steel can be considered a very basic valve material compared to other recent valve materials. This group of steels (patented in 1919 [10]) appears to be suitable for valve fabrication because it maintains its properties up to 600–650 °C under a high dynamic load [11]. Silchrome 1 has been designed and used since the 1920s and it is still in use today, available at the valve steel suppliers [12,13,14]. About a one hundred years of use demonstrates this material’s performance despite the fact that austenitic steels were also commercially available since the 1920s [12]. Nevertheless, the literature on Silchrome 1 and its use as a valve material, despite its long and very rich history, is sparse [10]. Only several studies cover the steel after being heat-treated completely, and the results are difficult to compare [7,11,15,16,17,18,19,20,21]. Moreover, it has to be taken into consideration that investigating historical samples faces problems linked to some level of inhomogeneity when comparing to modern materials. Due to less effective steel manufacturing processes in general, inclusions of a higher amount and larger size may be expected as well as perhaps some chemical composition inhomogeneity. This was found in many older studies, e.g., on the camshaft of a World War II era Volkswagen Kübelwagen, where, on the other hand, forging quality had been noted [22]. Banks [23] complains about the “considerable number of valve failures” due to defective material or variations in temperature during the manufacturing process, which he observed during his visits to the United States [23].

This paper aims at documenting Silchrome 1, a steel likely to have been employed for exhaust valves in the Harley-Davidson WLA/WLC motorcycle; revealing its properties; and pointing out the incidence of material evolution while also concerning the contemporary evolution in valve production tooling and procedures briefly in the context of the related vehicle. To identify the cause of a component’s failure, the knowledge of solved cases in connection with that being considered is highly beneficial. The same can be applied to metallurgy expertise. The work has been performed on an exhaust valve exemplar coming from a surplus unused replacement kit made in 1942 for a Harley-Davidson WLA/WLC motorcycle. This study starts with a valve description, followed by metallographic analysis, element composition and distribution assessment and hardness measurements. Then, the historical steel in its original heat-treatment condition is compared to the properties of its modern steel equivalent X45CrSi9-3 and heat treatment provided by the present standards: Valve steels and alloys for internal combustion engines and: Valve materials [24,25]. Standard-provided heat treatment was applied on historical material samples and compared to untreated original samples by hardness testing.

The aims of the paper can be formed into the following questions:

- Is the steel employed for the valve in 1942 close to the modern material standard [9]-grade one in terms of chemical composition?
- Is the original 1942 valve heat treatment in coherence with the steel modern material standard?
- Is the hardness, as the basic mechanical properties indicator, of the 1942 valve comparable with the steel modern material standard [24,25]?

## 2. Valve Manufacturing

**Figure 1:**
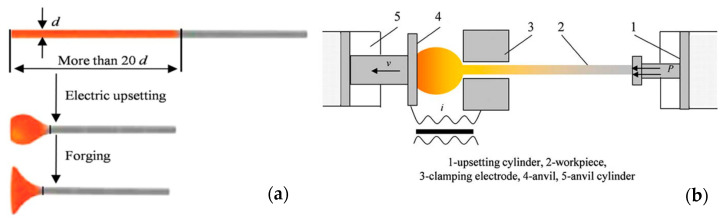
Schematic illustration of the valve head manufacturing (**a**) and valve electric upsetting process (**b**) [26].

In this paper, only the information with direct reference to the examined valve is displayed, as literature considering other valve types is abundant. When considering a single-piece, single-material valve, the valve head is shaped when hot as shown in Figure 1 [26]. This comprises three steps: cutting, electric upsetting and forging; see Figure 1a. A cold piece of bar cut to an appropriate length is fitted to the clamping electrode (central die) facing two moving counterparts: the left die (anvil) and the upsetting cylinders workpiece (hammer); see Figure 1b [26]. A changeable direct current, low-voltage and high-current electrical circuit between the die and the anvil is then set up and the part of the bar is heated by the Joule effect (Figure 1b) [26]. The process operates in the temperature range of 900–1000 °C to put the steel in the austenite range of stability. Plastic deformation occurs from the hammer’s axial pressure and the anvil’s slow backward movement, resulting in a ball-shaped future valve head, made in next die forging step [26].

**Figure 2:**
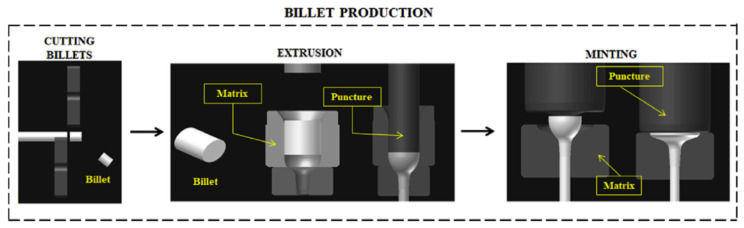
Schematic illustration of the valve head manufacturing process with billet extrusion [27].

Another way of achieving the desired shape of the head is the billet extrusion process illustrated by Figure 2, which operates according to the three following steps: cutting, extrusion and minting [27]. The first operation aims at cutting long metal bars into smaller pieces called billets at temperatures between 300 °C and 900 °C, according to the bar alloy [27]. Billets are subsequently extruded and minted by hydraulic press force in dies (matrix in Figure 2), while extrusion produces the valve fillet by employing a high level of deformation [27]. The minting subsequently shapes the valve head using an appropriate punch (mandrel) [27]. The whole recent valve production process includes many consequent steps: friction welding, face welding, machining, heat treatment, surface treatment, dimension checking, etc.

**Figure 3:**
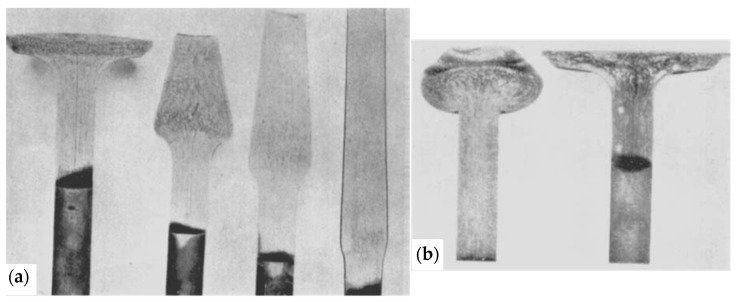
Heating and forming methods of valve heads: (**a**) gather upset and (**b**) electrical upset [23].

For the 1940s valves considered in this paper, however, the heating method appeared to be crucial. In the USA, two heating methods—gas heating and electrical induction heating—were employed in this period [23]. These are referred to as gather upset (Figure 3a) and electrical upset (Figure 3b) [23].

The final product quality and the number of consequent failures are considered similar [23]. It is obvious that, over the course of many years, electrical induction heating replaced the use of gas, and head shaping is generally performed in one or two following steps, which is possible even with large-diameter valves and nonferrous alloys [28,29].

## 3. Materials and Methods

### 3.1. The Investigated Valve

**Figure 4:**
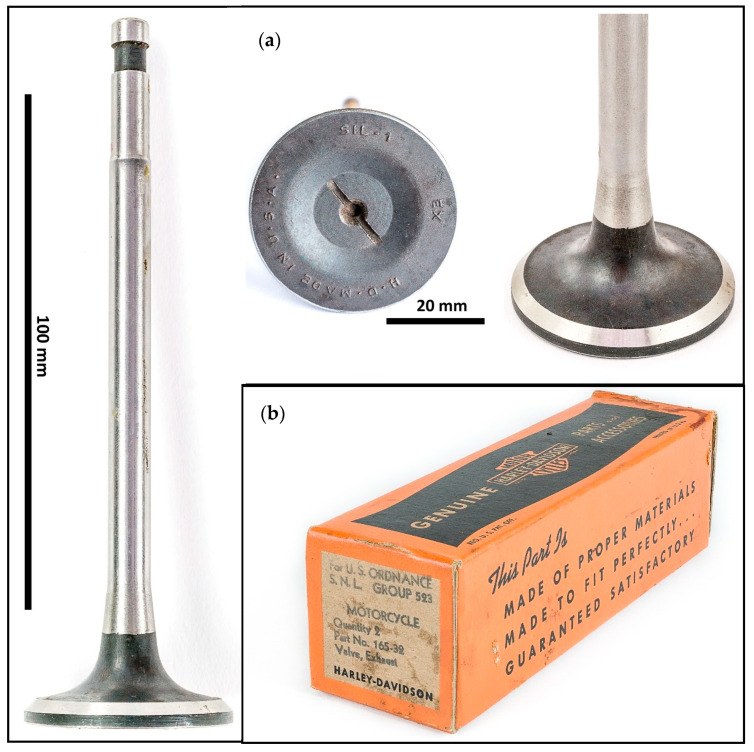
Harley-Davidson WLA 1942 motorcycle surplus exhaust valve (**a**) and its original box (**b**).

The investigated valve (Figure 4a) comes from a surplus unused replacement kit (2 valve pieces): Figure 4b, made in 1942 for a Harley-Davidson WLA/WLC motorcycle. Markings at the valve disc read H. D. MADE IN U. S. A. SIL-1 EX, indicating piece origin, material and application. The overall length of the valve is 142.15 mm, the disc diameter 41.00 mm and the stem diameter 8.62 mm.

### 3.2. Structural Observations

The entire valve referred to as HDEN1 (Harley-Davidson Exhaust New 1) was cut along the axial direction of the valve. Due to the valve shape, the main cut was performed by wire electrical discharge machining (WEDM) using a ZAP BP05dw device (Zakład Automatyki Przemysłowej B.P., Kutno, Poland). Moreover, this cutting method does not affect the mechanical properties of the cut piece. One of the valve halves was then cut into small pieces by ATM Brillant 220.2 (Verder Scientist, Haan, Germany) and Struers Accutom-100 (Struers, Rødovre, Denmark) standard automated laboratory saws. The other valve half was mounted entirely into a polymer resin to examine the grain flow (flow lines) and perform the hardness profile test later on.

The large specimen for hardness profile testing was polished with SiC paper and diamond pastes up to 3 µm, which in general was difficult. Inclusions in the polished surface were studied after polishing of the large specimen by hand on a Struers LaboPol-25 (Struers, Rødovre, Denmark) to 1 µm, whereas the small samples (described later in Section 3.5) were polished with an ATM Saphir 520 (Verder Scientist, Haan, Germany) automated polishing device.

Then, the large sample was etched with Vilella’s solution (3 g picric acid, 1 mL nitric acid, 1 mL hydrochloric acid and 100 mL ethanol) for 30 s, indicating the grain flow in macrophotographs. Vilella’s solution is commonly used with ferritic and martensitic steels, especially Fe-Cr, Fe-Cr-Ni and Fe-Cr-Mn alloys, for displaying carbides in quenched and tempered alloy steels, indicating grain boundaries in stainless steels, and is also used as a hot macro etchant in Fe-Cr-Ni and Fe-Cr-Mn steels [30,31,32,33]. Marble’s reagent (2.5 g CuSO_4_∙5H2O, 15 mL hydrochloric acid and 35 mL d. water) was used for 10 s, after repolishing, indicating a microstructure. Marble’s reagent is a general reagent and macro etchant for high-alloy, stainless and tool steels, commonly used for revealing structure, grain size examination in aged heat-resistant alloys and revealing carbides and intermetallic phases as well as being used as a hot macro etchant for Fe-Cr-Mn steels [30,31,32,34].

For the scanning electron microscopy aside from the Marble’s-reagent-etched specimen, one smaller cross-section sample of the head was prepared. In this case, polishing up to 0.25 µm was performed followed by polishing with Struers OP-U NonDry suspension colloidal 0.04 µm silica suspension, pH: 9.8 (Struers, Rødovre, Denmark) three times for 20 min, producing a very flat surface.

Light optical microscopy of the whole HDEN1 valve cross-section was performed on Zeiss Axioplan SIP42113, Zeiss AXIO Vert.A1 (Carl Zeiss Light Microscopy, Göttingen, Germany) and Keyence VHX-7100 microscopes (Keyence Corporation of America, Itasca, IL, USA), and samples for the heat-treatment experiment were examined on Nikon Eclipse MA200 microscope (Nikon Corporation, Tokyo, Japan). Image analysis was used to evaluate microscopical observations. The macrophotographs as well as Figure 4 were obtained using the Kaiser RS2CP light table (Kaiser Fototechnik GmbH & Co., Buchen, Germany) by Olympus OM-DE-M10 (Olympus Corporation, Tokyo, Japan) and Samsung NX5 cameras (Samsung, Seoul, South Korea).

A Hitachi S-3400N (Hitachi, Tokyo, Japan) scanning electron microscope (SEM) was employed for basic observations of the microstructure. Enhancing the crystallographic orientation contrast of the grains was performed by ECCI (Electron Channeling Contrast Imaging) applying the backscatter electron pole-piece detector in the Hitachi SU5000 (Hitachi, Tokyo, Japan). Among others, this method shows the grains’ crystallographic contrast by varying grayscale tone depending on each grain lattice orientation referring to varying electron backscatter intensity with the chemical contrast also present in the retrieved image [35,36]. A detector with enough spatial resolution as well as rigorous sample preparation, as mentioned above, is essential for this observation.

### 3.3. Chemical Composition Determination

Element quantitative analyses of the HDEN1 valve as well as element distribution were determined using a Cameca SX100 (CAMECA, Gennevilliers, France) Electron Probe Micro-Analyzer (EPMA) using four wavelength spectrometers (WDS). The analyses were carried out at 15 kV, 40 nA, using a PC2 crystal to detect the Ka X-ray of the carbon, a LiF crystal to detect the Ka X-rays of the nickel, chromium and manganese, a PET crystal to detect the Ka X-rays of phosphorous, sulfur and molybdenum and a TAP to detect the Ka X-rays of the silicon, aluminum and magnesium.

**Figure 5:**
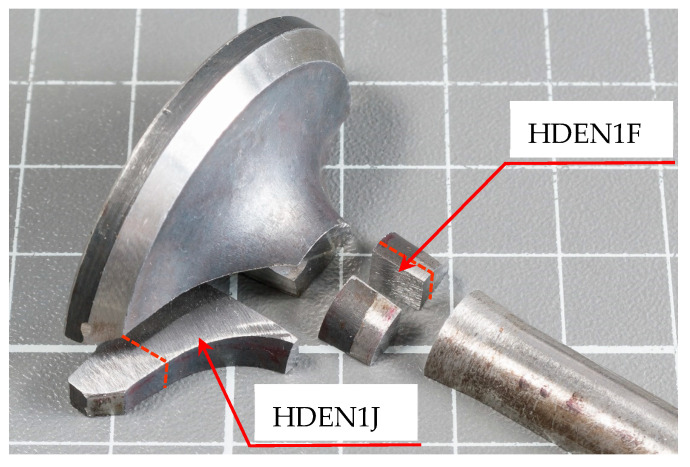
Sample location for the determination of valve chemical composition.

The steel’s element composition was determined with the sample HDEN1F (see Figure 5), on an average area of about 500 µm × 500 µm which included the matrix as well as small inclusions. The matrix composition itself was determined at two selected areas free of visible inclusions. Further carbon determination by Non-Dispersive Infrared Absorption (NDIA) using the CS744 Carbon/Sulfur Determinator (LECO Corporation, Saint Joseph, USA) was performed on the HDEN1J sample (see Figure 5).

### 3.4. Hardness Profile Test

**Figure 6:**
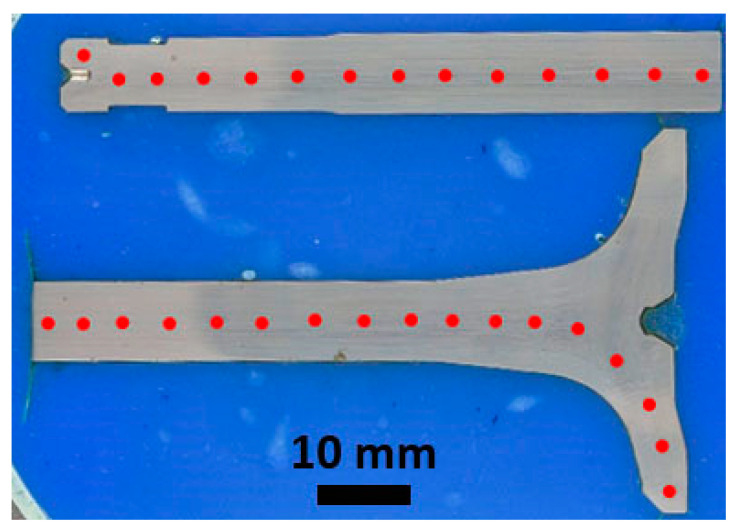
Approximate indent locations on the HDEN1 valve.

As mentioned previously, the HDEN1 valve cut lengthwise provided two identical halves. The first half, mounted entirely into a polymer resin, used for the macrophotographs and microscope observation, was also the subject for hardness profile testing. A Mitutoyo AVK C1 (Mitutoyo Corportaion, Kanagawa, Japan) hardness tester was used according to the Vickers hardness test HV1 standard (test force 9.807 N, duration of the test force 10 s) [37]. Tests were performed by indents starting on the tip of the stem, going through the valve stem and head to the face, step by step with ca. 5 mm spacing as shown in Figure 6. The obtained values refer to a general course of the hardness in the whole valve.

### 3.5. Heat Treatment–Hardness Experiment

**Figure 7:**
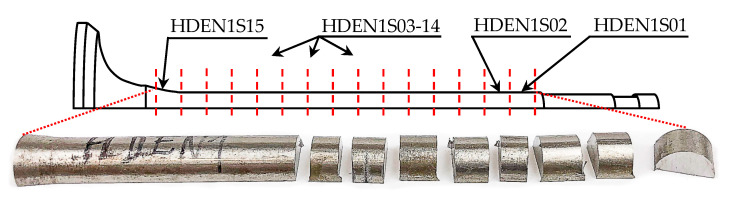
First half of the HDEN1 valve stem cutting scheme and labeling of cut pieces for heat treatment experiment. Letter “S” stands for stem.

The second half of the HDEN1 valve was cut perpendicularly to the valve axis into fifteen small parts about 5 mm × 8 mm in size for heat treatments and hardness tests as shown in Figure 7. The pieces to be heat treated according to Valve steels and Valve materials standards [24,25] were organized into three groups of four pieces each [24,25]. Each group was austenitized at either 1000, 1025 or 1050 °C for 30 min, followed by oil quench. For the consecutive tempering, four groups of samples were arranged, each by one from the previous group’s order. The first group was left as quenched and the others were tempered at 720, 770 and 820 °C for 30 min followed by air cooling.

All fifteen samples were mounted into resin supports and grinded and polished up to 1 µm after the heat treatment. Each sample was subjected to five regular hardness measurements and, in case of concerns, extra points were added, employing a Future-Tech FM700 tester (Future-Tech Corp., Kawasaki-City, Japan) according to the Vickers hardness HV1 test standard (test force 9.807 N, duration of the test force 10 s) [37].

## 4. Results and Discussion

### 4.1. Macro and Micro Structure Observation

**Figure 8:**
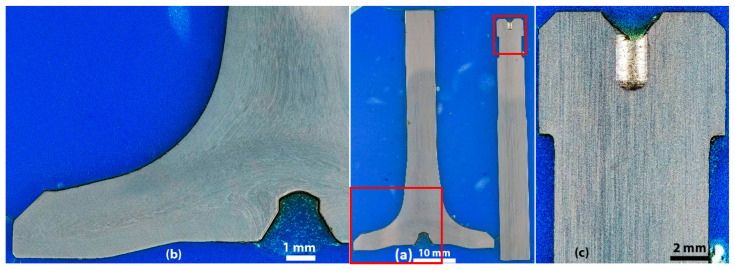
(**a**) Overview of the flow lines in the HDEN1valve revealed with Vilella’s solution and details in (**b**) the disc and (**c**) in the stem tip.

The macrostructure of HDEN1 valve etched by the Vilella’s solution is shown in Figure 8. The aligned marks in the upper part of the stem (Figure 8c) are probably simply the results of the bar forming before the valve fabrication. In the lower part of the stem (valve fillet) and in the valve disc (Figure 8b), the structural curved shaping marks or grain flow (flow lines) illustrate the forming of the valve head and can be compared with Figure 3.

Whether this valve head has been produced by gather upset or electrical upset is not easy to state and, therefore, it is left to the reader’s assessment. Undoubtedly, the tip of the valve was processed by conventional machining.

**Figure 9:**
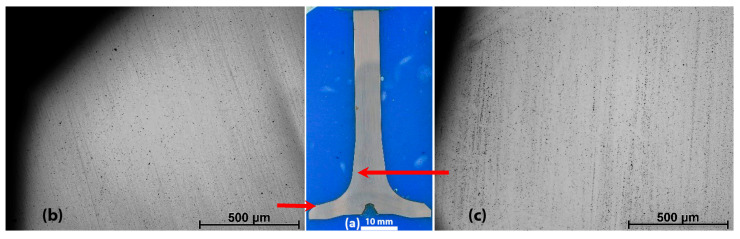
(**a**) Overview of the HDEN1 valve polished to 1 µm and details in (**b**) the head and (**c**) in the fillet; polished to 1 µm.

**Figure 10:**
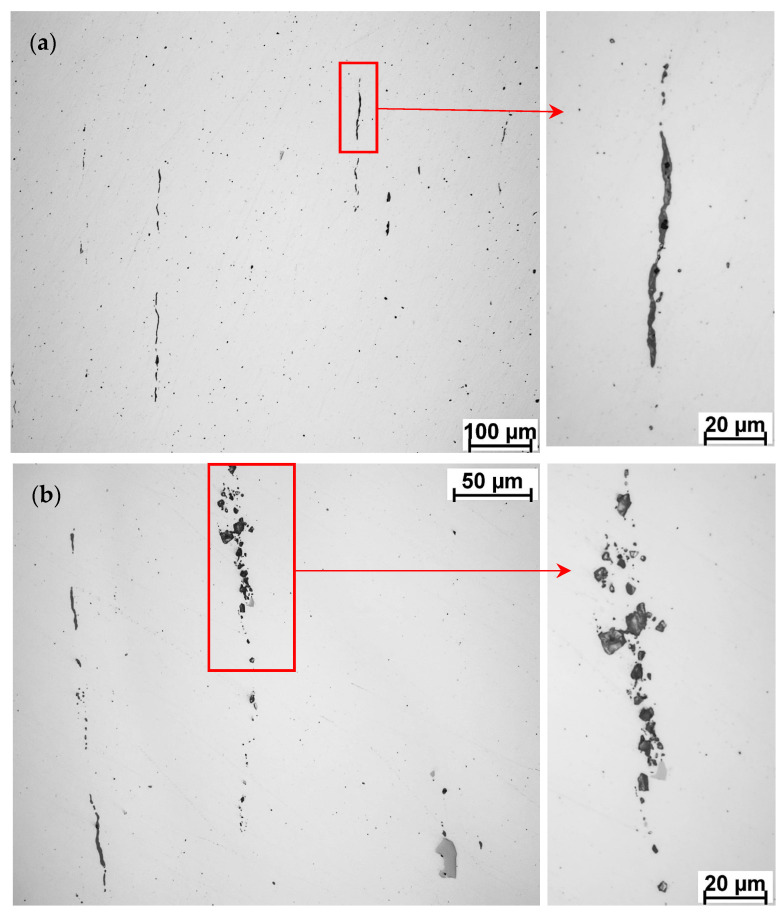
Line inclusions in the HDEN1 valve: (**a**) sample HDEN1S15 and (**b**) sample HDEN1S12; polished to 1 µm.

A large number of inclusions was observed all across the valve after polishing on non-etched surface under a light optical microscope (Figure 9 and Figure 10). Inclusions were aligned along the flow lines, in the shaping direction in the stem and in the fillet. In the valve disc, the line structure was more difficult to observe by point inclusion arrangement.

Inclusion of varying size (ca. 1–15 µm in diameter in general) contribute somehow to an overall hardness of the material, as they can never be avoided completely while placing the indents. The length of large individual line inclusions varied between ca. 100–900 µm, and they were located all alongside the stem with respect to the part’s shaping direction as well. During hardness testing, it was possible to avoid the large line inclusions (performed HV1 indent size in general ca. 70 µm diagonal) and therefore these did not affect the hardness measurement results. The significant number of inclusions was also a reason why the fine polishing of this material was difficult. Stem line inclusions of two different characters are shown on Figure 10. When sharp-edged particles become loose during the polishing, scratches are difficult to avoid, especially on the whole valve cross-section, where all the valve inclusions in the section plane are present. An overview of the inclusions’ chemical composition is displayed and discussed in the next chapter of this paper.

**Figure 11:**
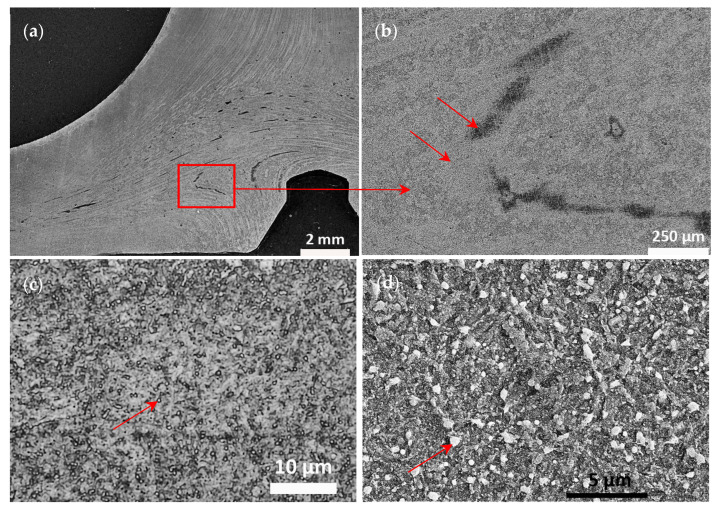
(**a**–**c**) Optical micrographs at different magnifications of the HDEN1 valve head etched for 10 s by Marble’s reagent and (**d**) secondary electrons—SEM image.

Etching the valve with Marble’s reagent revealed a uniform structure in the whole valve profile, with alternating bright and dark lines according to the shaping direction. Therefore, a general uniform heat treatment and a general uniform hardness over the valve could be expected. However, in the valve head, dark or bright areas (depending on the lightening) can be found, suggesting an expected inhomogeneity of the material; see Figure 11a,b. Nevertheless, the structure of these areas is identical when observing high-magnification images. However, individual grains were difficult to distinguish (Figure 11c), similar to the studies by Voorwald et al. [7] and Azadi et al. [15] also investigating Silchrome 1 steel [7,15]. Even at higher magnification with the SEM, neither prior austenitic grains nor a typical feature of the martensite structure, laths, were revealed (Figure 11d). However, it was possible to distinguish rather round particles of the same character as observed by Wu et al. [20] in their X45CrSi9-3 modern steel example [20]. They identified them as iron- and chromium-rich precipitates by SEM-EDS analysis and determined them to be (Cr, Fe)_7_C_3_ carbides by XRD analysis [20]. M_23_C_6_-type carbides that were not identified as present by Wu et al. [20] were investigated in depth by Liu et al. [38]; however, this was in a different type of martensitic steel, 5Cr15MoV steel [38].

**Figure 12:**
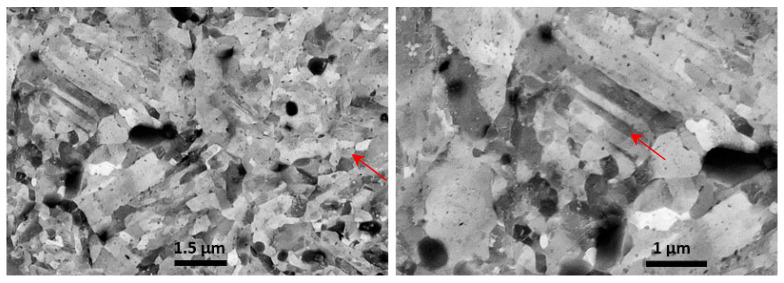
SEM-BSE ECCI image of HDEN1 valve head structure, polished 3 × 20 min OP-U ND.

The microstructure observed by optical microscopy at a higher magnification (Figure 11c) was compared to the Japanese studies from 1934 and 1942 [16,17,18]. There appears to be some similarity with the structure of steel with the following composition: C 0.406, Si 2.81, Mn 0.39, P 0.017, S 0.003, Cr 12.89 Mo 0.90, Cu 0.07 wt%, austenitized at 1050 °C for 1 h, quenched in oil, tempered for 1 h at 850 °C, leading to a hardness of 402 HV after quenching and 248 HV after tempering [17]. The observed microstructure agrees with tempered martensite, as shown, e.g., by Vander Voort et al. [30] and Machek et al. [39], which can also be referred to as sorbite [30,39]. To reinforce the description of this microstructure, the steel was observed in SEM-BSE ECCI mode on a very fine polished surface, see Figure 12 (preparation described in Section 3.2).

Fine, round, rather equiaxial grains were mostly present; however, there were also rare grains with a typical martensite-shaped structure. The displayed structures do not agree, however, with the observations of Saranraj et al. [19], where grain size of ca. hundred micrometers and appropriately sized carbides were found [19]. But a different etchant (HNO_3_:Di. H_2_0 1:3) and only light optical microscopy were used in their study [19].

### 4.2. Chemical Analysis Determination

The determined chemical composition of the investigated HDEN1 valve with comparison to contemporary sources and several standards is displayed in Table 1.

The measured composition of the HDEN1 valve matches the Silchrome 1 X45CrSi9-3 steel modern standard as shown in the last line of Table 1. Interestingly, it differs from the standard provided by Banks [23], as he indicates more silicon and less chromium compared to the ISO standard. In other standards, as displayed in Table 1, molybdenum is present. Mo, W and Al are the alloying elements suppressing the high-temperature tempering brittleness caused by excessive content of phosphorus, and the 0.15–0.25 wt% Mo content has a major sufficient effect [39]. Since the carbon content was not possible to be quantified by the EPMA-WDS, NDIA analysis was used instead.

Equivalent Japanese standard (Table 1), followed in the 1930s and 1940s, varies substantially in the content of chromium and brittleness of the valves had been reported [16,17,18]. However, Kuge [16] uses a Silchrome steel with a lower chromium and silicon content [16].

**Figure 13:**
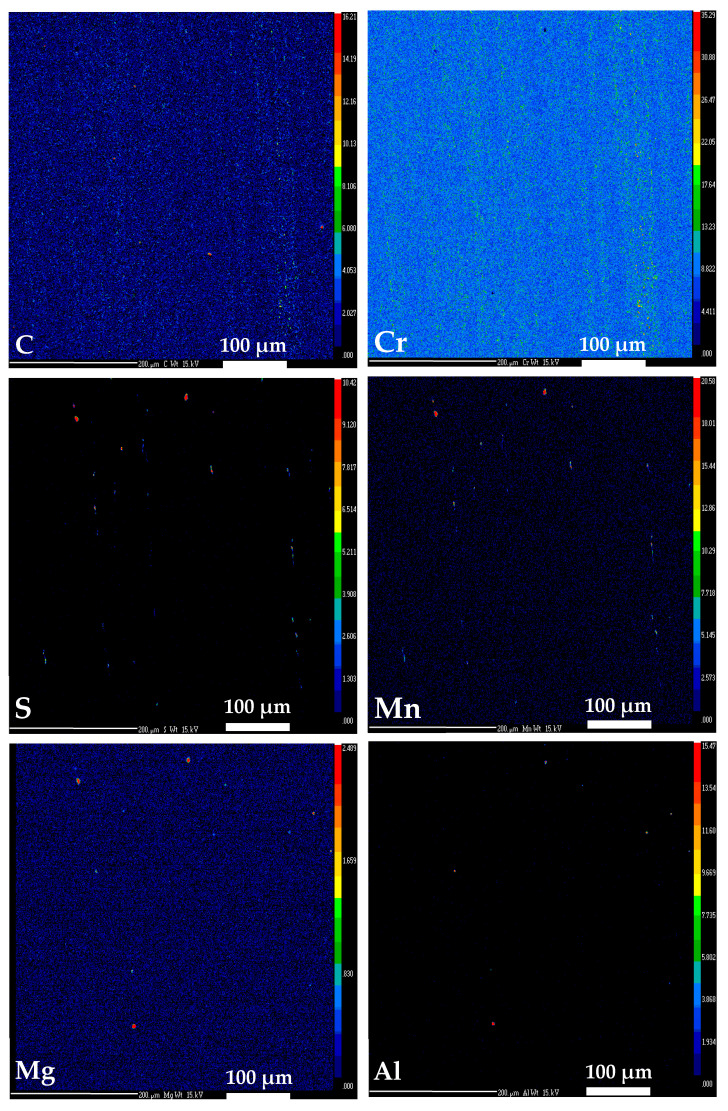
Chemical element mapping of HDEN1F sample by EPMA-WDS analysis, color scale ranging from dark blue, low content, to bright red, high content.

Worth mentioning is that phosphorus and sulfur content is slightly above all standards. This can be due to the fact that small inclusions were present in the examined area (ca. 500 µm × 500 µm). As can be seen in Figure 13, Mn-S-rich particles and Mg-Al-rich particles imaged by EPMA explain a higher sulfur content than the standard as well as the presence of aluminum and magnesium, respectively. Increased content of sulfur might have a positive influence on valve tribological properties [40]. Large inclusions (Figure 10) were not taken into selection for element composition analysis, as they can be avoided as mentioned earlier.

Despite that, there is no significant difference in element contents against the pure matrix; however, carbon and chromium distribution differs over the field of view, as it was found on the examined area (ca. 500 µm × 500 µm, Figure 13).

Segregation bands formed by these two elements distribution correspond with the direction of the part-shaping process, flow lines and inclusions arrangement. Despite the band phenomenon at the selected area not being represented by strong color contrast, it is indicated and in coherence with the microstructure inhomogeneity shown on Figure 8 and Figure 9.

### 4.3. Valve Hardness Profile

Hardness profile values, collected in Table 2, show that the hardness does not vary along the valve.

However, the first six values can be considered as slightly lower than the rest of the valve. This can be a consequence of the heat-treatment process and not due to simple scattering. It might be caused by the time delay of handling hot parts prior to quenching or uneven parts heating, as Banks [23] indicates gas furnaces as the main heating tools in all hot production steps [23]. Apart from that, the hardness profile suggests that, in general, the heat treatment was uniform all across the valve in coherence with the uniform microstructure.

Even though the average value of hardness 385 ± 16 HV obtained on the 1942 valve is a rough parameter, in this case it shows significant difference from the hardness indicated by the standards, i.e., 280–345 HV [24,25]. Worth noting is that these values are gained after ca. 80 years of storage. About 40–60 HV more than the DIN standards’ top value on average is, however, not so significant of an increase compared with the data published by Voorwald et al. [7]. In their paper, a hardness value of 345/446 HV was measured on the steel austenitized at 1040 °C followed by oil quenching and tempering at 680 °C [7]. While the DIN standards recommend to quench from 1000–1050 °C and to temper at 720–820 °C, this would suggest a non-negligible impact of the tempering temperature. In contrast, Jaswin et al. [41] quenched from 975 °C and tempered at 650 °C, reaching 345 HV [41]. And Kuge [16] with a steel of slightly different composition, shown in Table 1, obtained hardness values of 484, 327 and 266 HV by oil quenching from 1050 °C followed by tempering at 600, 750 and 900 °C, respectively [16]. Taking into account the experimental results and the data from the bibliography, it can be roughly estimated that the tempering has been performed at 680–720 °C.

### 4.4. Heat Treatment–Hardness Experiment

The hardness measured on the untreated material is collected in Table 3. The hardness partition is rather homogeneous, and a difference of 10 HV1 in general hardness average compared to the values shown in Table 2 is low.

The hardness values reported in Table 4 for heat-treated samples clearly indicate that hardness is very dependent on the tempering temperature but not on the austenitizing temperature.

The highest austenitizing temperature 1050 °C with the lowest tempering temperature 720 °C led to the highest hardness of about 341 HV, which satisfies the standard requirements (280–345 HV) [24,25]. Taking into account the values scattering, this is also true for the two other austenitizing temperatures and the same tempering temperature of 720 °C. In contrast, this is about 40–50 HV under the original valve’s hardness. To summarize, heat treatment prescribed by the standards [24,25] did not lead to the original 1942 condition of the Silchrome 1 steel HDEN1 valve [24,25].

Tempering temperature as well as tempering time must be also taken into consideration. In the present paper, 30 min tempering time was employed as the samples were very small in contrast to 60 min in the work by Jaswin et al. [41] and Voorwald et al. [7], who, respectively, employed 650 °C and 680 °C achieving 345 and 345/446 HV [7,41]. Nishigori [17] investigated the tempering time ranging from 15 to 240 min and found no difference in hardness with tempering times of 30 min and 60 min [17]. Kuge [16], in coherence with our paper, performed 40 min austenitization and 30 min tempering [16].

Petsov [11] showed that the hardness varied from 318 to 471 HV depending on level of deformation: 75–145% [11]. He used 550/650 °C tempering, however, for 60–180 min and austenitizing temperature was not specified [11].

Nevertheless, comparing with the literature is problematic, as the level of deformation is not indicated and it is unknown at the HDEN1 valve as well.

Finally, based on the previous paragraphs, a very rough estimation of the tempering temperature could be made as being in the range of 550 °C to 680 °C. Then, an interesting comparison with the Motorcycle Mechanics Handbook published in 1943 [42] could be made, where the representative temperatures diagram indicates the exhaust valve temperature as being between 650 °C to 760 °C [42]. That means the valve operation is at higher temperatures than the tempering value and this appears as a curious contradiction. Mascarenhas et al. [21], performing high-temperature accelerated wear tests with a counterpart low-alloyed steel (34CrNiMo6) seat, noticed slight valve hardness drop after a 750 °C service test; however, no microstructural change was reported [21]. Their study concluded there to be high wear damage of the valve face at ca. 730 °C, which corelates with the mentioned technical standards’ maximum designated temperature of 700 °C for Silchrome 1 [21,24,25].

## 5. Conclusions

The paper aimed at documenting the microstructure of an exhaust valve fabricated in 1942 for a Harley-Davidson WLA/WLC motorcycle and at comparing the material features with current steels. For that, an unused valve coming from surplus was subjected to metallographic analysis, heat treatments and hardness measurements.

The main conclusions and the answers to the questions in the introduction are as follows:

- Answer to the first question: The steel employed for the fabrication of the valve is Silchrome 1 steel in coherence with the X45CrSi9-3 steel modern material standard [9] but containing 0.3 wt% of molybdenum in the historical valve, which is not required by the ISO standard. As well, there is a slightly higher content of phosphorus and sulfur exceeding the standard limit, which can be connected with inclusions.
- The manufacturing process led to flow lines accompanied by inclusions which corresponds with the year of production of the part, and an inhomogeneous distribution of carbon and chromium was documented.
- Whether this valve head was produced by gather upset or electrical upset could not be determined and had been left to the readers’ assessment.
- Answer to the second question: The heat treatment–hardness experiment demonstrates that the original heat treatment is not in coherence with the steel modern DIN standard practice [24,25]. Most likely the tempering temperature was lower for the investigated valve in the surplus part, which contrasts with the service temperature indicated in the contemporary motorcycle mechanics handbook [42].
- Answer to the third question: The hardness of the surplus 1942 valve is comparable with the steel modern material standard and the historical valve hardness is, in general, even ca. 40–60 HV higher than DIN standard top value after ca. 80 years of storage [24,25].

## Figures and Tables

**Table 1:** Chemical composition of HDEN1 valve steel (carbon determined by NDIA, rest by EPMA-WDS) and comparison with standards and other papers.

Sample/Material	Chemical Composition (wt%)										
	C	Si	Mn	P	S	Cr	Ni	Mo	Al	Mg	Fe
**HDEN1F**	**0.455**	**3.19**	**0.51**	**0.06**	**0.05**	**9.08**	**0.45**	**0.30**	**0.07**	**0.10**	**Bal.**
Banks [23], 1938 Silchrome 1 Standard	0.40–0.50	3.50–4.25	0.40–0.60	max 0.04	max 0.03	7.50–8.50	0.50	-	-	-	Bal.
Yamanaka et al. [18], 1942 Silchrome sample A	0.40	3.50	0.45	0.017	0.010	12.40	-	-	-	-	Bal.
Yamanaka et al. [18], 1942 Silchrome sample B	0.36	3.04	0.43	0.022	0.007	11.57	-	-	-	-	Bal.
Nishigori [17], 1934 Silchrome charge 20254	0.406	2.81	0.39	0.017	0.003	12.89	-	0.90	-	-	Bal.
Nishigori [17], 1934 Silchrome charge 41356	0.451	2.49	0.30	0.020	0.006	12.68	-	1.00	-	-	Bal.
Kuge [16], 1942 Silchrome Styria sample	0.340	2.48	-	-	-	10.18	-	0.73	-	-	Bal.
Nishigori [17], 1934 Silchrome Standard	0.30–0.45	2.00–3.30	max 0.60	max 0.030	max 0.030	9.00–13.00	-	0.70–1.3	-	-	Bal.
4Kx9C2 Standard	0.35–0.45	2–3	max 0.8	max 0.03	max 0.025	8–10	max 0.6	max 0.3	-	-	Bal.
40Kx10C2M Standard	0.35–0.45	1.9–2.6	max 0.8	max 0.03	max 0.025	9–10.5	max 0.6	0.7–0.9	-	-	Bal.
Wu et al. [20], 2015 Commercial X45CrSi9-3	0.41	2.24	0.28	-	-	9.25	0.08	-	-	-	Bal.
X45CrSi9-3 Standard [9,25]	0.40–0.50	2.70–3.30	max 0.60	max 0.040	max 0.030	8.00–10.00	max 0.50	-	-	-	Bal.

**Table 2:** Hardness profile values along the HDEN1 valve.

HDEN1				Tip	Stem												
Measurement No.				1	2	3	4	5	6	7	8	9	10	11	12	13	14
Mitutoyo AVK C1 HV1				371	372	377	383	371	369	382	389	379	391	391	388	391	387
**Stem**									**Fillet**				**Disc**				**Avg.**
15	16	17	18	19	20	21	22	23	24	25	26	27	28	29	30	31	-
387	391	385	393	391	396	396	393	397	389	390	384	373	382	388	377	381	385±16

**Table 3:** HDEN1 valve reference piece hardness, original condition.

Hardness Tester	Sample	Heat Treatment	Polish	HV1					
				1	2	3	4	5	Average
Mitutoyo.	**HDEN1S01**	Original	**1 µm**	391	391	393	394	396	393 ± 3
Future-T.				401	406	405	404	403	404 ± 3
Mitutoyo AVK C1				402	451	403	402	399	411 ± 40
	**HDEN1S06**	Original	**1 µm**	381	374	384	388	380	381 ± 7
				383	380	389	385	387	385 ± 5
				387	389	384	387	381	386 ± 5
	**HDEN1S11**	Original	**3 µm**	406	385	390	392	398	394 ± 12
				404	397	443	404	413	412 ± 31
				390	394	390	397	395	393 ± 3
-									396 ± 17

**Table 4:** Effect of heat treatment on HDEN1 valve sample hardness.

Hardness Tester	Sample (1 µm Polish)	Heat Treatment (°C)		HV1					
		Quenching	Tempering	1	2	3	4	5	Average
Future-Tech FM700	**HDEN1S02**	**1000**	**-**	742	747	764	743	744	748 ± 16
	**HDEN1S03**		**720**	333	334	333	331	331	332 ± 2
	**HDEN1S04**		**770**	304	311	303	300	303	304 ± 6
	**HDEN1S05**		**820**	296	298	299	296	301	298 ± 3
	**HDEN1S07**	**1025**	**-**	773	771	769	761	766	768 ± 7
	**HDEN1S08**		**720**	335	338	343	335	336	337 ± 6
	**HDEN1S09**		**770**	308	312	313	311	306	310 ± 4
	**HDEN1S10**		**820**	297	292	290	289	293	292 ± 4
	**HDEN1S12**	**1050**	**-**	718	659	666	770	785	719 ± 66
	**HDEN1S13**		**720**	336	339	342	342	347	341 ± 6
	**HDEN1S14**		**770**	313	306	311	310	306	309 ± 4
	**HDEN1S15**		**820**	293	294	291	289	289	291 ± 3

## Data Availability

Data are contained within the article.
